# Interaction and psychological characteristics of art teaching based on Openpose and Long Short-Term Memory network

**DOI:** 10.7717/peerj-cs.1285

**Published:** 2023-03-21

**Authors:** Chen Qi

**Affiliations:** School of Art, Sichuan University Jinjiang College, Meishan, Sichuan, China

**Keywords:** Computer vision, Multimodal analysis, Teaching evaluation

## Abstract

As living standards improve, people’s demand for appreciation and learning of art is growing gradually. Unlike the traditional learning model, art teaching requires a specific understanding of learners’ psychology and controlling what they have learned so that they can create new ideas. This article combines the current deep learning technology with heart rate to complete the action recognition of art dance teaching. The video data processing and recognition are conducted through the Openpose network and graph convolution network. The heart rate data recognition is completed through the Long Short-Term Memory (LSTM) network. The optimal recognition model is established through the data fusion of the two decision levels through the adaptive weight analysis method. The experimental results show that the accuracy of the classification fusion model is better than that of the single-mode recognition method, which is improved from 85.0% to 97.5%. The proposed method can evaluate the heart rate while ensuring high accuracy recognition. The proposed research can help analyze dance teaching and provide a new idea for future combined research on teaching interaction.

## Introduction

With the development of artificial intelligence technology, the continuous change of traditional teaching methods, and the popularization of online and mixed teaching, art teaching has also undergone significant changes. It is becoming more and more common to give lectures in person and video lectures. With the development of deep learning and machine learning (Fotiadou et al., 2002), teaching efficiency and flexibility are also greatly improved. For example, in vocal music teaching, through natural language processing technology, the student’s pronunciation and intonation are analyzed to complete the refined analysis of vocal music teaching; Similarly, in dance teaching, through the recognition of human posture and intelligent computing to complete the fine division of dance movements, the skill level of dancers can be rapidly improved.

Art is an emotion (Jun et al., 2020). Whether painting, dance, or vocal music art, it is a means for human beings to express their feelings. Through the study of art, we can significantly cultivate our sentiments and help people regulate their thoughts and feelings. Therefore, we should pay more attention to ways and methods in art teaching and constantly deepen the reform of art teaching. Through more diversified means, we should develop all sides, from teaching methods, thinking and skills to teaching art (Inomjonovna, 2023). While developing exemplary art teaching, we should also consider the psychological changes in the teaching process. We can only get twice the result with half the effort by mastering students’ learning rules and psychological state. Taking dance teaching as an example, it is a bilateral teaching activity that requires the construction to carry out the unification and standardization of dance movements jointly. Students’ reception ability and psychological quality are involved in the teaching and teaching content. The psychological process of three-dimensional continuous flow in dance teaching activities has a specific structure and systematic laws (Kalyani et al., 2019). In dance teaching, students’ psychological state significantly impacts their reception ability, not only in completing current learning tasks but also in the subsequent development of dance thinking. Dance is the most direct, thorough and complete form of expression. When the students are in good psychological condition and their imagination is more abundant, they can abstract and refine the phenomenon higher than life in the dance movement and form a new image in their brain, which belongs to the category of psychology. Therefore, in the teaching process, we should not only master the completion of students’ teaching tasks but also need to know more about students’ mentality (Liu & Zhang, 2021; Hong & Yingzi, 2020).

Online and mixed teaching courses are usually recorded through video images. Therefore, given the existing equipment and teaching environment, there is an urgent need for a new teaching evaluation method to understand the student’s learning situation and consider their psychological conditions. In video teaching evaluation, video stream information includes spatial and temporal dimension features. Therefore, obtaining effective spatiotemporal dimension features becomes the focus of video field research. Given the achievements of convolutional neural networks in the image field, how to introduce a convolutional neural network into the video field has become the focus of many researchers. A 3D convolutional neural network is a video action recognition algorithm emerging in recent years, including the 3D convolutional network (C3D), the pseudo 3D reactive network (P3D) and the influenced 3D convolutional network (I3D) (Tran et al., 2015; Qiu, Yao & Mei, 2017). Compared with the traditional 2D convolution neural network, the convolution core of the 3D convolution neural network is a cube rather than a square, which enables the convolution core to extract the spatiotemporal characteristics of video frames directly.

On this basis, people complete the work of extracting human motion features and motion posture information from video streams. In the analysis of the mental state, people usually use EEG and ECG technology to complete cognitive state assessment and emotional calculation. However, such equipment is expensive and cumbersome for dance art teaching. It is acceptable to conduct small-scale research, but applying it to large-scale education is difficult. Some emotions, such as tension and relaxation in work, study and life, can be expressed through heart rate (Vrijkotte, van Doornen & de Geus, 2000). With the continuous development of wearable devices, it is possible to use heart rate observation to analyze people’s tension. At the same time, long-term heart rate monitoring can also judge the physical fitness of the human body, which also plays a vital role in dance teaching. Shi et al. (2019) proposed an approach to student behavioral movement recognition based on Fisher Broad Learning System (FBLS). They defined seven classroom behaviors: head-turning, hand-raising, reading, dozing off, listening, writing, and standing. Fang et al. (2011) designed a gesture recognition system for students, using image segmentation technology to identify student objects. According to the relationship between similar object areas, students’ behaviors were defined as right-hand raising, left-hand raising, hands raising, prone lying, standing and normal. Huang et al. (2020) used deep convolutional neural networks (D-CNN) and cascade facial feature point positioning methods to recognize students’ classroom behavior. Cheng et al. (2020) proposed a student behavior recognition method based on a deep convolution generation adversarial network. Shukor et al. (2018) analyzed students’ sitting posture using pressure sensors and connected students’ sitting posture with their attention in class. Zuraini et al. (2020) adopted the K-means algorithm to monitor the heart rate to understand students’ behavior.

Therefore, to meet the needs of teaching actions evaluation and psychological state analysis in dance art teaching. This article takes art dance teaching as the research object and uses existing video technology and wearable devices to evaluate dance teaching methods. In the actual teaching, due to the occlusion of images or personal privacy, the video cannot completely show the students’ movement status, so the wearable device heart rate data is used to complement each other to complete the high-precision recognition of students’ dance movements while completing the evaluation of psychological emotions to a certain extent. In the method proposed in this article, dance video data is used to extract motion features, the TCN network is established, and state judgment is formed. At the same time, the dancer’s state is recognized according to the collected heart rate data. Finally, it is fused with image recognition data to form an adaptive decision-making model, complete dance action recognition, and help teachers complete the analysis of the psychological state.

## Methodology

**Figure 1:**
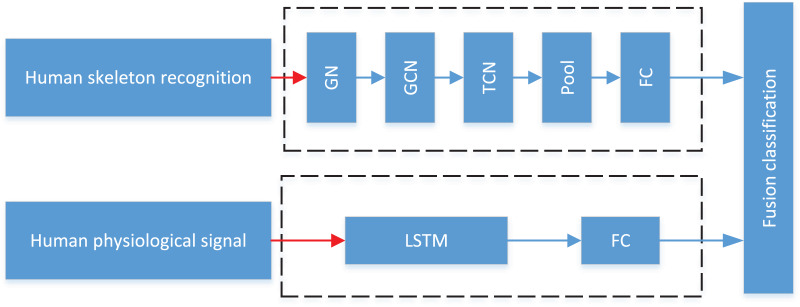
The framework of the fusion classification. The overall process is shown: first, the Openpose framework is used to preprocess RGB video data, extract the human skeleton, and the skeleton feature map is used to complete action recognition through graph convolution network and time convolution network.

In practical application scenarios, the single-mode data recognition method has a high rate of false recognition due to the influence of occlusion or signal loss. To meet the needs of students’ action in art teaching, this article combines computer vision technology and ECG heart rate recognition to jointly evaluate the students’ psychological characteristics and activities. The overall process is shown in Fig. 1, where GN represents Graph Network; GCN means Graph Convolutional Network; TCN defines Temporal Convolutional Network; FC describes Fully Connected: First, the Openpose framework is used to preprocess RGB video data, extract the human skeleton, and use the skeleton feature map to complete action recognition through graph convolution network and time convolution network; also, through the bracelet worn by the students, the heart rate characteristics are monitored and the joint analysis is completed through LSTM network.

### Skeleton sequence extraction and network structure construction based on Openpose

First, we should preprocess their original video images to ensure students’ privacy in art teaching. According to the need for action evaluation and action observation in art teaching, this article selects the Openpose framework to take the lead in extracting skeleton frames (Cao et al., 2017). The Openpose framework proposes a joint point affinity field to solve the occlusion problem between objects and people. In essence, it is a two-dimensional vector field set containing the position and direction information of the limbs. Using the position information and PAFs of each joint point of the skeleton, it calculates the correlation degree of the critical nodes and completes the subsequent human model splicing. The central network of Openpose can be summarized by Formulas (1) and (2):

(1) $${S^t} = {\rho ^t}(F,{S^{t - 1}},{L^{t - 1}}),\forall t \ge 2$$

(2) $${L^t} = {\varphi ^t}(F,{S^{t - 1}},{L^{t - 1}}),\forall t \ge 2$$

where F is the feature map obtained through the convolution network first, $\rho$ and $\varphi$ both represent networks. *S*_*t*_ is the joint point confidence map output at *t*, and *L*_*t*_ is the affinity of the two pairs of relationship nodes output at *t*, namely, the weight coefficient. To ensure network convergence, the method uses the L2 loss function for the loss functions of both branches. To avoid gradient disappearance, only the output of the last layer is used in the prediction process. The loss values at each stage are shown in Formula (3):

(3) $$f_s^t = \sum\limits_{i = 1}^J {\sum {W(p) \bullet \left\| {S_i^t(p) - S_i^*(p)} \right\|} } _2^2$$

(4) $$f_L^t = \sum\limits_{j = 1}^J {\sum {W(p) \bullet \left\| {L_j^t(p) - L_j^*(p)} \right\|} } _2^2$$

(5) $$f = \sum\limits_{t = 1}^T {(f_s^t + } f_L^t)$$

where the one with superscript * represents the actual value, the one with superscript *t* represents the predicted value at different stages, *p* represents each pixel point, *W (p)* represents the missing mark at this point, and its value is 0 or 1. If it is 0, the loss value is calculated, as shown in the overall loss value Formula (5).

**Figure 2:**
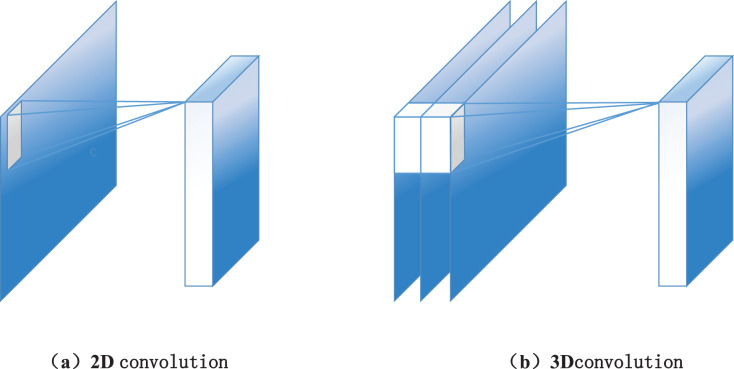
The diagram of (A) 2D convolution and (B) 3D convolution. The network structure layer is shown: After the graph is convolved, pooling is performed, and finally full connection design is performed.

After the human skeleton extraction based on Openpose, the 3D map convolution technology is used to complete the network construction of motion features. The 3D convolutional neural network is a deep learning algorithm widely used in video action recognition. Compared with the convolutional neural network algorithm in the image field, its essential difference is that the two-dimensional convolution core in the image field is “expanded” into a three-dimensional convolution core. Its schematic diagram is shown in Fig. 2. The reason for choosing a 3D convolutional network is that the video stream data contains the spatial dimension information within each data frame and retains the information between frames. Because of the particular convolution kernel structure of 3D convolutional neural networks, it can learn spatial and time dimension features. Similar to the use of an image convolution network, during the training and use of a 3D convolution neural network, the original video data can be directly used as the input of the network instead of having to extract video frame data and optical flow data. Compared with the 2D convolutional neural network, 3D convolutional neural network significantly improves parameters because of the “expansion” of time dimension on the convolution core.

The convolution process of graph map convolution adopted in this article is shown in Formula (6):

(6) $${f_{out}}({v_{ti}}) = \sum\limits_{{v_{ti}} \in B({v_{ti}})} {\displaystyle{1 \over Z}} \,\,fe{a_{in}}({v_{ti}}) \cdot \omega ({l_{ti}}({v_{ti}}))$$

where ${f_{out}}({v_{ti}})$ the graph convolution is output at the node *v*_*ti*_, *v*_*t*i_ is the frame of node *i* in the skeleton node sequence at time t, which represents the adjacent set of node *v*_*ti*_ with K subsets, Z is the node feature of *v*_*ti*_, and *l*_*ti*_ represents the corresponding mapping of B to K-1 data, namely convolution. The network structure layer is shown in Fig. 2. After the graph is convolved, pooling is performed, and finally, complete connection design is achieved.

### Motion recognition based on the heart BPM

As for the heart rate signal collected by wearable devices, its accuracy is limited. The frequency domain features such as FFT, wavelet transform and Hilbert Huang transform can hardly produce the expected observation effect due to the noise problem (Wang, 2021). Therefore, according to the actual situation of the collected signal, only the time domain signal is analyzed. The commonly used statistical features for a single channel time domain signal include mean, extreme, peak, *etc*. On this basis, the sliding variance of the sliding window is used as the feature, and its calculation method is shown in Formula (7):

(7) $$\sigma _{{c_i}}^2 = \displaystyle{1 \over {2w + 1}}\sum\limits_{j = i - w}^{j = i + w} {{{\left( {{c_j} - {{\overline c }_j}} \right)}^2}}$$

where $\sigma _{{c_i}}^2$ represents the sliding variance result of heart rate, *c*_*j*_ is the heart rate value collected at the *j* point, *w* is the width of the sliding window, ${\overline c _j}$ is the mean value in the *j* point window. In this way, the size of the sliding window w can be changed to adapt to the size and length of the action, thus ensuring the algorithm’s accuracy to a certain extent.

After feature extraction is completed, the pattern shown in Fig. 1 is used for recognition. Due to the solid temporal nature of dance movements, the LSTM method is used for the decision-level classification of time series in this article. Compared with the traditional RNN method, the LSTM method can effectively solve the problem of long-term memory dependence due to the existence of forgetting gates (Formula 8). In this article, the network input is a five-dimensional statistical feature, mainly including: mean, extreme value, skewness, standard deviation and sliding variance.

(8) $${f_t} = \sigma ({W_f} \cdot [{h_{t - 1}},{x_t}] + {b_f})$$

where ${h_{t - 1}}$ represents the hidden node status, $W$ is the linear matrix and $b$ is biased; σ represents the activation function Sigmoid.

### Fusion classification

To ensure the optimization of model identification, this article adopts an adaptive multimodal decision fusion method (Nakano et al., 2020). The classification scores calculated by 2.1 and 2.2 are defined as ${e_{t,1}}$ and ${e_{t,2}}$ respectively, and the prediction vector formed is as follows in Eq. (9):

(9) $${E_t} = {[e_{t,1}^T,e_{t,2}^T]^T}$$

The calculation is carried out according to the label vector Jj in the training process. The goal is to maximize the probability in Eq. (10) through the weighted weight *W*_*i*_ of both. At this time, the result generated by the iteration is optimal.

(10) $$\arg \mathop {\max }\limits_{{W_i}} {W_i}{E_t}{J_j}$$

*W*_*i*_ becomes the optimal weight matrix, similar to the voting mechanism. The two types of optimal results are fused through the optimal weight to achieve optimal identification under data fusion.

## Experimental Results and Analysis

### Data processing and feature extraction

**Figure 3:**
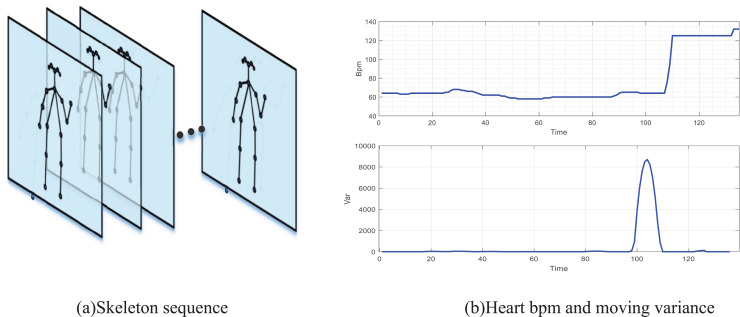
Processed video sequence and heart bmp. (A) The extraction of human motion skeleton framework based on Openpose, 17 skeleton segment models are used in this article, and the original video data is used to form a human motion skeleton video stream through Openpose network. At the same time, the results of heart rate acquisition through the bracelet wearable device are shown in (B).

In this article, according to the characteristics of dance teaching in art teaching, four main dance movements in school teaching are selected for recognition, and video recording equipment is set up in the particular classroom. This article uses an RGB network camera to collect video data, and heart rate is monitored through the bracelet. As shown in Fig. 3A, the extraction of the human motion skeleton framework based on Openpose, 17 skeleton segment models are used in this article. The original video data form a human motion skeleton video stream through Openpose Network. At the same time, the results of heart rate acquisition through the wearable bracelet device are shown in Fig. 3B. This article intercepts the heartbeat data of the bracelet wearer from preparation to the first action, and it can be seen that there is an obvious step in their heart rate. In contrast, in the sliding variance feature, it can also be seen that there is a significant change in the sliding variance at the beginning of the action. Therefore, the selected features are suitable for the dancer’s motion recognition.

**Figure 4:**
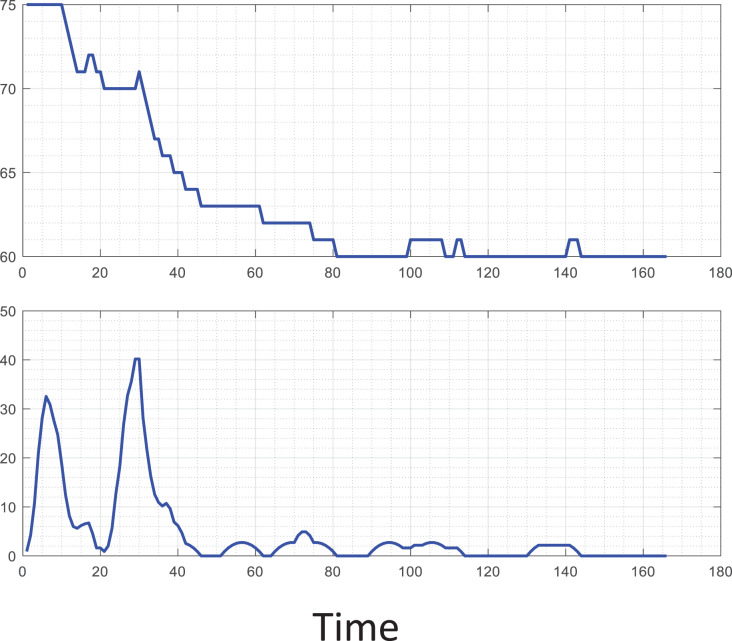
Typical heart bpm analysis. The heart rate characteristics and variance changes of the new student after completing some actions. During the experiment, observing the expression of the student can clearly find the tense frown.

At the same time, to reference the analysis of typical signals in teachers’ teaching, this article analyzes the specific signals of tension, as shown in Fig. 4. It shows the new student’s heart rate characteristics and variance changes after completing some actions. During the experiment, observing the expression of the student can find a tense frown. After completing the activity, the heart rate gradually recovers and has more fluctuations. Therefore, we can focus on the data fluctuating violently in future emotional analysis.

After the model is built, the network model shown in Fig. 1 is used for training. The k-fold method divides the original data into K groups (k-fold), makes a verification set for each subset, and the rest of the K-1 subset data as a training set. In this way, K models can be obtained. Each of the K models evaluated the results in a validation set. Because the deep learning method requires much overhead for sample learning and optimization, this article does not use the K-fold method in traditional machine learning. Still, it divides the training and test sets into 70% and 30% proportions. The classification results of the proposed model are discussed in detail in the next section.

### Classification result

To verify the effect of the adaptive fusion method proposed in this article, in addition to mutual recognition, we also use video and heart rate data to recognize dance movements and compare some leading indicators in machine learning. The recognition indicators used in this article are the precision P, recall R, and F1 score (Yacouby & Axman, 2020). The multi-classification problem in this article also uses the three parameters of the extended version; that is, when evaluating a specific action, this action is regarded as a positive category, and the rest of the actions are regarded as negative categories.

**Figure 5:**
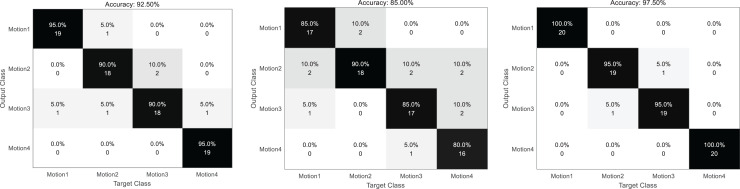
Confusion matrix of three classification results. From left to right: the confusion matrix of the results of video separate classification, heart rate separate classification and their joint classification.

The confusion matrix formed by the model prediction results is shown in Fig. 5. From left to right are the confusion matrix of the results of separate video classification, heart rate different classification and their joint classification. The darker the color is, the higher the recognition rate is. It can be seen that the collaborative classification model proposed in this article has a better effect, and its classification results are relatively concentrated. Except for some confusion between actions 2 and 3, all the others complete the classification correctly. The heart rate method is used for poor classification, which is consistent with the initial expectation. Because the heart rate method has a single mode and the signal accuracy is limited, its recognition result is poor. At the same time, in the process of dance movement, the change of heartbeat is continuous, and more confusion in the continuous part is also based on the significant disadvantage of single-mode signals.

**Figure 6:**
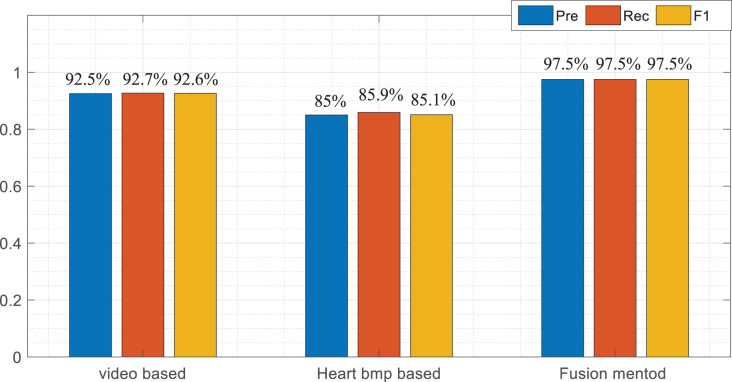
Result comparison among multimodal data. In order to more intuitively illustrate the classification effect among the three models, this article calculates the accuracy rate, property right rate and F1 score of the three models respectively.

To more intuitively illustrate the classification effect among the three models, this article calculates the accuracy rate, property right rate and F1 score of the three models, respectively. By comparing the index shown in Tables 1–3 and Fig. 6, we can find that the proposed classification fusion model performs better than the single-mode recognition method in all aspects, while the action recognition method in the video mode is almost the same as the fusion method in some actions. This shows that the video signal class accounts for a large proportion of the information fusion process, consistent with the Wi mentioned in 2.3.

**Table 1:** Video-based classification. We can find that the proposed classification fusion model performs better than the single-mode recognition method in all aspects.

	Motion1	Motion2	Motion3	Motion4	Mean
Pre	95%	90%	90%	95%	92.5%
Rec	95%	90%	85.7%	100%	92.7%
F1	95%	90%	87.8%	97.4%	92.6%

**Table 2:** Heart BMP based classification. The action recognition method in the video mode is almost the same as the fusion method in some actions.

	Motion1	Motion2	Motion3	Motion4	Mean
Pre	85%	90%	85%	80%	85%
Rec	89.5%	75%	85%	94.1%	85.9%
F1	87.2%	81.8%	85%	86.5%	85.1%

**Table 3:** Fusion classification. The video signal class accounts for a large proportion in the process of information fusion, which is consistent with the *W_i_* mentioned in 2.3.

	Motion1	Motion2	Motion3	Motion4	Mean
Pre	100%	95%	95%	100%	97.5%
Rec	100%	95%	95%	100%	97.5%
F1	100%	95%	95%	100%	97.5%

## Discussion

Recently, it has been essential to upgrade teaching methods, teaching contents and teaching evaluation methods in an all-around way through new technologies. In art teaching, for example, vocal music teaching uses natural language processing technology to complete speech recognition, and dance teaching uses video image analysis technology to identify dance movements to form a refined evaluation. Video image technology has developed rapidly, and the number of contributions to top-level computer conferences such as CVPR and ICCV has increased year by year, becoming the most important research hotspot in the computer field. Based on this, this article categorizes and recognizes common actions in art dance teaching through video stream data. At the same time as technological development, more attention should be paid to students’ mental health. Especially in the current new pandemic, everyone’s activity area and social experience are limited, which should pay more attention to psychological counseling in teaching.

In teaching, especially in art teaching, we should combine the law of students’ psychological growth, master the art of teaching methods and methods skillfully and creatively, organize teaching creatively, and integrate basic knowledge, basic technology, basic skills, and sports psychology with aesthetics; The content of dance movements is characterized by elegance and artistry, which requires high physical quality, and to some extent, causes certain learning difficulties for some students. In the process of teaching, the positive artistic mood of adjusting teaching mood and paying attention to teaching is a weak, calm and long-lasting emotional state, which has a pretty long duration. The teaching mood affects the teaching behavior of teachers and students and will make everything around them infected with this emotion. In teaching, some games will be interspersed. If the distribution is improper, it will cause psychological pressure on students and affect the teaching mood. Only in a positive and good teaching mood can teachers make teaching become a vibrant teaching whole full of vitality and vitality through reasonable organization and distribution, which has a soul-stirring and shocking artistic charm. The guidance of self-confidence and the establishment of teachers’ praise, encouragement and psychological suggestion can arouse students’ self-confidence, stimulate their potential and meet students’ needs for love and respect. The rational use of motivation and strengthening the art of teaching motivation can fully mobilize students’ enthusiasm for learning. Teachers can also use praise as an auxiliary teaching evaluation art. The so-called evaluation art refers to the PE teachers’ assessment of students’ mastery of teaching content and the teacher’s recognition of students. It is essential to assess each student with respect appropriately. How to evaluate poor students and improving their academic performance should be the teaching focus of PE teachers. Teachers must teach these students patiently in the principle of care, love and encouragement, and pay attention to the improvement process in evaluating their learning effect so that these students can see their progress and enhance their self-confidence.

Of course, achieving the above teaching art means understanding the students’ mentality. Therefore, this article uses wearable bracelets to monitor the students’ heart rates based on the current equipment characteristics to achieve action recognition in dance teaching and learning. On the one hand, it makes up for the shortcomings of video signal blocking and signal instability; on the other hand, it hopes to evaluate the students’ psychological state through heart rate monitoring. By comparing the natural heart rate with the standard and relaxed heart rate, we can analyze their psychological tension and make predictions about their physical fitness to achieve a win-win situation for mental and physical fitness evaluation. The adaptive fusion recognition method combined graph convolution network and LSTM model used in this article has a higher longitude. Mutual recognition is more robust than the traditional convolution network and the different learning methods based on machine learning. At present, psychoanalysis based on heart rate can only conduct a preliminary study on the psychological state; that is, it can judge the most fundamental tension and relaxation state and cannot achieve emotional calculation. With the development of science and technology, it will have a better effect to conduct a deeper multi-mode fusion analysis of video data with portable EEG, ECG and other signals.

## Conclusion

This article proposes an adaptive fusion classification model framework that integrates video technology and heart rate signals. The Openpose network is used to take the lead in extracting human skeleton motion features to ensure privacy. The extracted signals are formed into new video stream data, and the graph convolution network is used to complete the probability identification of actions. At the same time, the LSTM network is used to complete the probability identification of the heart rate data the other way, and the adaptive weight learning method is used to achieve the optimal classification decision. By comparing the classification results, the multi-mode fusion method used in this article has a higher recognition accuracy. At the same time, by observing the actual heart rate signal and the sliding variance signal, the psychological state of the dancers can be analyzed to realize the joint analysis of multimodal information in the teaching process and ensure the teaching effect. As single-mode information, the stability of heart rate signal and the impact of psychological state analysis are challenging to meet the needs of accurate analysis. Therefore, we hope to combine more modal information in future research to form a more precise analysis.

## Supplemental Information

10.7717/peerj-cs.1285/supp-1 Supplemental Information 1 Code.

## Additional Information and Declarations

### Data Availability

The following information was supplied regarding data availability: The code is available in the Supplemental Files. The data is available at Zenodo: Chen Qi. (2023). Data Set for Interaction and Psychological Characteristics of Art Teaching based on Openpose and LSTM Network [Data set]. Zenodo. https://doi.org/10.5281/zenodo.7528453.
